# The challenges of communicating research evidence in practice: perspectives from UK health visitors and practice nurses

**DOI:** 10.1186/1472-6955-12-17

**Published:** 2013-07-09

**Authors:** Jennifer E van Bekkum, Shona Hilton

**Affiliations:** 1 MRC/CSO Social & Public Health Sciences Unit, University of Glasgow, Glasgow, UK

## Abstract

**Background:**

Health practitioners play a pivotal role in providing patients with up-to-date evidence and health information. Evidence-based practice and patient-centred care are transforming the delivery of healthcare in the UK. Health practitioners are increasingly balancing the need to provide evidence-based information against that of facilitating patient choice, which may not always concur with the evidence base. There is limited research exploring how health practitioners working in the UK, and particularly those more autonomous practitioners such as health visitors and practice nurses working in community practice settings, negotiate this challenge. This research provides a descriptive account of how health visitors and practice nurses negotiate the challenges of communicating health information and research evidence in practice.

**Methods:**

A total of eighteen in-depth telephone interviews were conducted in the UK between September 2008 and May 2009. The participants comprised nine health visitors and nine practice nurses, recruited via adverts on a nursing website, posters at a practitioner conference and through recommendation. Thematic analysis, with a focus on constant comparative method, was used to analyse the data.

**Results:**

The data were grouped into three main themes: communicating evidence to the critically-minded patient; confidence in communicating evidence; and maintaining the integrity of the patient-practitioner relationship. These findings highlight some of the daily challenges that health visitors and practice nurses face with regard to the complex and dynamic nature of evidence and the changing attitudes and expectations of patients. The findings also highlight the tensions that exist between differing philosophies of evidence-based practice and patient-centred care, which can make communicating about evidence a daunting task.

**Conclusions:**

If health practitioners are to be effective at communicating research evidence, we suggest that more research and resources need to be focused on contextual factors, such as how research evidence is negotiated, appraised and communicated within the dynamic patient-practitioner relationship.

## Background

Evidence-based practice and patient-centred care are two important aspects of UK Government policy reforms to improve the delivery of healthcare [1-4]. However, communicating best evidence alongside respecting individual patient preferences can pose a challenge to health practitioners, particularly when evidence is contested or the weight of evidence is unclear [5-7]. Evidence-based practice and patient-centred care stem from very different paradigms [5,8]; the former from positivistic philosophy and the latter from humanistic philosophy. As Rycroft-Malone et al. (2004) state: “the craft of mixing the scientific with the human presents very real challenges, particularly if these do not fit together well” [9] p.86.

In the past, the production of evidence and its implementation into practice has been viewed as a positivistic and linear process [10]. More recent work in this area has tried to integrate positivistic and humanistic paradigms by recognising the complex, multifaceted and sometimes ‘messy’ nature of evidence and its application as an evolving body of facts, value judgements, experiences, and interpretations, which are contingent on time and context [9,11,12]. From a more integrated perspective, moving evidence into practice is a shared endeavour involving individuals, multidisciplinary teams, organisations, and the wider policy context [10,13]. But at the individual level, health practitioners who work in direct contact with the public play a pivotal role in communicating about the latest research evidence. Communication skills are known to be an important factor in narrowing the gap between evidence-based practice and patient-centred care, and in improving patient health outcomes [14-17]. As such, health practitioners can be described as ‘frontline facilitators’ of evidence implementation into practice; how well they communicate this evidence with patients depends, in part, on their appropriate skills, attributes and knowledge. Importantly, evidence-based information needs to be communicated in a clear and understandable way that enables patients to make informed decisions [18]. However, it is widely recognised that many barriers exist that can impede practitioners’ effective communication of research evidence to patients, such as a lack of: time; organisational support; authority; and critical appraisal skills [19-23].

Although there is a body of research addressing communication within healthcare [15,24-27], limited research has focussed on the challenge of communicating evidence to patients in the context of the community setting, such as general practitioner (GP) surgeries and health centres. These settings can be particularly challenging when evidence emerges that conflicts with current practice, as these health practitioners are often the first point of contact for patients and people seeking information and reassurance about health issues. Redsell and colleagues [28] used semi-structured interviews (n = 22) to investigate health visitors’ communication strategies for endorsing childhood immunisations to parents. A key finding was that health visitors discussed a loss of professional confidence in the wake of the measles, mumps and rubella (MMR) vaccine crisis and that their normal communication models were not sufficient to deal with parents’ anxieties. Hilton et al. [23] also investigated health visitors’ use of research evidence regarding child health issues using a survey study design (n = 185). Out of the health issues addressed, health visitors reported having the least confidence in searching for and communicating information about childhood immunisations, and many reported conflicting evidence as a barrier to using research in practice.

Our present study follows on from this work [23] and forms part of a larger research project called Communicating Health Information and Research into Policy and Practice (CHIRPP), which focuses on health professionals' engagement with research evidence. This paper presents a descriptive qualitative account of how health practitioners in community practice settings negotiate the challenges of communicating health information and research evidence in practice.

## Methods

### Sampling and recruitment

Eighteen in-depth telephone interviews were conducted with health visitors (n = 9) and practice nurses (n = 9) between September 2008 and May 2009. Participants for this study were recruited across the UK via adverts on the Royal College of Nursing website (n = 6), posters advertising the study at the 2007 Community Practitioners and Health Visitors Annual Conference (n = 8), and through recommendation (n = 4). Convenience sampling was used to obtain a diverse range of practitioners in terms of: age; length of experience in the health service; type of caseload; and geographical location within the UK (see Table 1). Both health visitors and practice nurses were identified as suitable professions to be included in this study as they work with a broad range of publics and deal with a wide variety of health issues. Additionally, both these practitioners work within settings in the community as part of multi-disciplinary medical teams but often work autonomously with patients on a one-to-one basis. Practice nurses’ daily work may involve monitoring and providing: information on a range of chronic conditions; wound care; health screening; and family planning, as well as delivering vaccinations and running health promotion interventions. They generally carry out their work from GP surgeries and health centres. Health visitors’ daily workload involves running clinics and carrying out home visits to offer parental support and information on infant and family health. This may include advice on: weaning; feeding; dental health; developmental screening and health checks, and delivering childhood vaccinations. (http://www.nhscareers.nhs.uk/explore-by-career).

**Table 1:** Participant demographics

**ID no.**	**Age**	**Sex**	**Length of service (yrs)**	**Area/caseload**	**Regions UK**
HV01	60	F	22	Deprived, city	London
				High ethnic population	
HV02	32	F	6	Affluent, city	South East England
				High alternative types population	
HV03	53	F	29	Mixed, city	West Midlands
				High ethnic population	
HV04	49	F	12	Mixed, city	South West England
				High ethnic population	
HV05	4	F	22	Affluent, city	South East England
HV06	63	F	27	Mixed, rural	Scotland
HV07	47	F	16	Mixed, rural	South East England
				High alternative types population	
HV08	61	F	36	Mixed, rural	South West England
HV09	52	F	26	Mixed, rural	Scotland
PN01	39	F	6	Deprived, rural	East Anglia
PN02	34	F	8	Mixed, rural	North East England
PN03	44	F	19	Mixed, city	London
PN04	36	F	2.5	Mixed, city	South West England
PN05	28	F	7	Affluent, city	South East England
PN06	55	F	30	Deprived, rural	Scotland
PN 07	50	F	8.5	Mixed, rural	Scotland
PN08	59	F	20	Mixed, city	West Midlands
PN09	49	F	17	Affluent, city	Yorkshire and Humberside

### Data collection and analysis

Ethics approval for the study was granted from the NHS National Research Ethics Committee. All of the interviews were carried out over the telephone by SH: 14 participants while at their home and four while at their workplace. Informed consent was obtained from all participants prior to interview. Interviews lasted approximately 40 minutes. The use of telephone interviews was a particularly appropriate data collection method. This was not only a convenient method for contacting and talking to busy practitioners, but yielded rich data as practitioners were able to speak openly about the challenges they faced [29].

The interview schedule was semi-structured and exploratory, allowing for flexibility in the questioning to enable the researcher to develop an in-depth understanding of the topic under study [30]. The interview schedule included five broad themes: demographic details (e.g. patient caseloads and service length); sources of information that they currently use; conflicting evidence; confidence; and assessing research evidence. Probes were used to encourage participants to talk about the health issues that they commonly deal with, such as: vaccines; weaning; feeding; and new medical treatments. At the time these data were collected, it was one decade after Wakefield and colleagues’ [31] publication that suggested a link between the MMR vaccine and autism, and a large body of evidence had subsequently emerged that refuted Wakefield’s claims. However, at the time of these interviews, Wakefield’s paper had not yet been retracted, nor had he been struck off of the medical register, and uptake of the MMR vaccine was still lower than before the controversy hit the headlines in 1998. It is also important to note that UK policy and training for health visitors and practice nurses has undergone some substantial changes since the current study was undertaken. Nevertheless, there is no reason to believe that the challenges that practitioners spoke of experiencing in their daily jobs have been entirely resolved with this training.

The interviews were audio recorded and transcribed verbatim. To ensure anonymity, participants were assigned an individual code and all names were removed from the transcripts. Each transcript was then read and re-read by JvB and SH to identify tentative patterns and themes within the data [32]. Once all the transcripts had been thoroughly read, principles of constant comparative method were used to develop and refine the themes further [33]. The next stage in the process, carried out by JvB was to use Inspiration software to map out the potential links between the data and dominant discourses, and to ensure that all relevant data were included in the themes. With the developed data maps, the study’s researchers took part in a ‘depth perception’ exercise [34] to help create a more critical analysis into the participants’ accounts by asking ‘why’ questions about the content, and suggesting ideas that had not been included in the findings. This process led to minor changes in theme secs and content to ensure the theme secs were clearly representing the data content and to ensure that there was minimal overlap of data between themes.

## Results

In this section, three main themes are presented. These capture participants’ perceptions of ‘communicating evidence to the critically-minded patient’, ‘confidence in communicating evidence’, and ‘maintaining the integrity of the patient-practitioner relationship’.

### Communicating evidence to the critically-minded patient

A dominant theme that emerged from the interviews was the challenges that participants faced when communicating about evidence with an increasing number of critically-minded patients. Critically-minded patients were commonly described by participants as: ‘educated’; ‘affluent’; ‘assertive’; ‘research-oriented’; ‘well-informed’; and very capable of using the Internet for sourcing information. One health visitor commented that: “…in the more affluent areas, people are very much research focused and they will look on the Internet, they will look at all the alternatives…” (HV08). Across the interviews, participants spoke of a wider cultural change in their relationship with patients, which involved patients becoming more proactive in assessing evidence themselves and less accepting of ‘blanket’ (HV 05), ‘one size fits all’ (PN02) health advice. For instance, as one practice nurse suggested: “There is a kind of new era where, you know, our parents’ generation wouldn’t have questioned us as health professionals, but now more and more people are becoming questioning, because people go to university and feel more confident in themselves, confident in their own work lives and they’re professional” (PN09). Similarly, another practice nurse spoke about practitioners generally having less influence, stating: “…even doctors aren’t just, aren’t just like you know, accepted what they say as gospel anymore, people are encouraged to find things out for themselves really now” (PN04).

Some participants drew on the MMR vaccine controversy to illustrate the communication challenges they experienced when patients questioned and disputed evidence. The participants often discussed how the most dissent about the vaccine was voiced by more affluent parents. Although this new wave of patients were viewed as being well informed and savvy, participants spoke of the troubles they experienced trying to communicate evidence-based information to parents who were basing their beliefs on personal and social influences as opposed to the existing body of research evidence. As one health visitor explained: “During the MMR controversy the evidence was unclear and parents often based their judgements on the views of friends and family, and that kind of evidence can be very persuasive” (HV06). Other participants also spoke about the role of anecdotal or experiential evidence. It was mentioned that when people base their decisions on experiences rather than on research evidence it raises difficulties for participants who champion evidence-based practices. As one health visitor stated: “How do you say that’s not fact, it’s one person’s experience”’ (HV02). Similarly, a practice nurse suggested that people rely on different forms of evidence, stating: “…it can be quite different to different people” (PN07). It was common for participants to describe patients becoming more assertive about their healthcare decisions. The exception to this, mentioned by some participants, was that patients from deprived communities were: “…less educated and less likely to question the care or information they were given, they tend to just say ‘yes, it’s good for the child’ and they’ll do whatever you suggest” (HV01).

### Confidence in communicating evidence

Another strong finding that emerged from the data was the impact that more critical and questioning patients were having on the participants’ confidence to communicate best evidence. Participants illustrated this by references to feeling ‘inadequate’ when talking to ‘more educated parents’ about research evidence, and to difficulties that arose when parents and patients have read extensively around specific topic areas (HV02, PN06). Some participants described feelings of embarrassment and they used words such as “stupid” **(**HV08, PN01**,** PN09), “silly” (PN01), and “a complete idiot… a twit” (PN04) to describe how they felt about their level of skill in assessing evidence. Participants spoke of how dealing with knowledgeable patients could impact negatively on their own self-esteem. As one health visitor explained: “It actually makes you feel quite de-skilled, because you end up thinking […] they’re much better than me, and I can’t add anything to this conversation” (HV07).

Another issue that emerged was that participants often felt patients have unrealistic expectations about the depth and breadth of health visitors’ and practice nurses’ knowledge. For example, one practice nurse said: “There are so many areas that we cover, patients sort of challenge you. They expect you to know everything” (PN04). It was common for participants to feel that they did not have a good understanding of the strengths and limitations of different types of evidence. They talked about feeling pressured by patients to know more, as this quote illustrates:

I have had some parents, sometimes, questioning me about evidence to the extent to where I say, “I don’t know, for God’s sake, don’t ask me things like that.” They’re asking me about, you know, the constitution and the make up of vaccines, and I think, you know, I know a reasonable amount and I could find out for them, but sometimes you just think – I’m not a bloody scientist. What do you expect me to know? […] You know you feel almost like you are under attack (PN09).

There was a sense from all the interviewees that the more confident the participant is about understanding the strengths and limitations of evidence, the more likely they feel they are to have a positive impact on patients’ understandings.

### Maintaining the integrity of the patient-practitioner relationship

Managing and maintaining long-term positive relationships with patients, often referred to in literature as a therapeutic relationship, also emerged as a key theme. Many participants felt that managing their relationships with patients carefully was crucial to facilitating effective communication of research evidence. Throughout the interviews, participants often stressed the importance of building trust with patients. As one health visitor commented:

There is a fine balance between advocating something that you have strong views about but also recognising their view. That’s the beauty of health visiting because that’s the whole way you work, getting a relationship going with the client and you might be trying to say something which they don’t want to hear but it’s only through time and trust, building a relationship, then they might not necessarily change what they do but they will hopefully hear what you’re saying […] we’re gonna be saying some things which people don’t want to hear but […] and you’ve got to have a relationship with your clients that you can still get into the house again (HV07).

Participants commonly spoke of how building a trusting relationship with patients was the foundation to effectively communicating about evidence. A couple of participants recalled specific examples to highlight this point. One health visitor spoke of a successful relationship that she had nurtured over several years:

She was a single mother and trusted me. I think that’s really important. I worked with her to get the information across, so you know, we work together because otherwise people get disengaged and then there’s nobody left there to work with them (HV08).

Not having the necessary information at hand to be able to answer patients’ queries was thought, by some participants, to undermine patients’ trust in them. For instance, one practice nurse stated: “If they think that you don’t know what you’re talking about, then they’ll have no trust in you…” (PN01**).** When probed about what kinds of things they do to strengthen their relationship with patients, it became evident that developing a long-term relationship tended to be achieved by taking a non-directive approach when speaking about evidence. From many of the participants’ accounts provided, persuading patients to accept evidence-based practices was secondary to maintaining the integrity of the patient-practitioner relationship. Being non-directive was associated with “being careful about what you say” (PN08), “not doing anything to harm the relationship” (HV05), and “knowing when to back off” (HV07). For example, one health visitor explained the use of a less directive approach as a means to avoid alienation when giving advice:

You can see sometimes, you know, some mothers will just switch off from what you’re saying, they won’t come back to the baby clinic […] because they, you know they don’t agree with that advice that’s being given. Clients are all different […] you look, you see how your advice is being received really cos there’s no point giving it if it’s not being received (HV05).

Only one participant, who had over 20 years of practice experience and worked in a deprived community setting, discussed using a more directive, paternalistic approach when talking with patients to ensure research evidence was clearly communicated and adhered to. She made no mention of this approach being detrimental to her relationship with patients (HV01).

## Discussion

Our findings complement previous work that has explored health practitioners’ experiences of communicating evidence [23,28]. The current findings provide novel insights into how health practitioners negotiate some of the challenges that can impede them from effectively communicating and using research evidence with patients. This work also highlights some of the documented tensions that exist between evidence-based practice and patient-centred care [6,18] within the context of the community practice setting. In summary, health visitors and practice nurses within this study believed that communicating research evidence is a hugely important but challenging aspect of their job, in part due to the complex and dynamic nature of evidence and the changing knowledge, attitudes and expectations of patients. They raised issues about: increasingly critically-minded patients who would question and dispute best evidence; the detrimental effect that these patients with increasing knowledge and healthcare expectations were having on their professional confidence; and the challenge of communicating best practices while effectively managing their relationship with patients.

Wider socio-cultural conditions, often discussed in terms of late-modernity and the rise of consumerism, are proposed to underpin the changing nature of patients and their increasing knowledge and expectations about healthcare [35-39]. According to Seale [39], individuals now tend to live, work and travel away from their communities and receive information from multiple sources of expertise, made easily accessible through mediums such as the Internet. He considers that in a social climate that offers little security, individuals are compelled to make more critical choices about their identity, which involves a growing preoccupation with risk evaluation. Therefore, people are actively constructing their identity through a more reflexive and self-determining process than perhaps was done in the past and are more inclined to question authority and trust, which is reflected in attitudes towards the medical profession [39,40]. As a result, power imbalances and the knowledge gap that once distinguished the health professional from the lay person have radically diminished [41,42]. It is some of these changes, seemingly more prevalent in affluent and Internet savvy patients, which health practitioners in this study found daunting and disempowering. According to McMullan [37] healthcare professionals who feel threatened by the information and knowledge patients present during their encounter are more likely to communicate in a practitioner-centred way by defending their expert opinions. This goes against the policy move towards patient-centred care and self-management [1,4,43]. Many of the health practitioners in this study were acutely aware of the importance of maintaining a good relationship with their patients and facilitating them to make their own decisions. However, our participants sometimes struggled with the tension of communicating best evidence while not damaging the integrity of their longer-term relationships with patients, which they feared could result in disengagement and poorer patient outcomes.

Communicating with critically-minded patients about evidence-based practice proved a greater challenge for practitioners when evidence was contested within the public domain. Although public access to health information is expanding, this poses challenges in knowing who to believe and trust. Health practitioners in this study were unsure of the level of weight to give second-hand or third-hand accounts of evidence, such as media reports or re-told stories from friends or family that are often readily available. Tools to assist health practitioners to discuss the merits and pitfalls of such sources of evidence may be a useful resource.

The findings also highlight the lack of clarity, on the health practitioners’ part, about how to contextualise patients’ personal and social influences and experiences with existing evidence-based guidelines. This echoes the ongoing debates within the literature about what types of knowledge constitute evidence and how to integrate these different types of knowledge into guidelines and practice [7]. It has been argued that information that is of personal value and importance to patients should be considered alongside best evidence [18]. Health professionals are recommended to move past static, one-way communication of evidence and towards an appreciation of the personal, social and political influences attached to any healthcare decision [44]. Entwistle et al. [45] suggest that information from personal experiences can be used to complement general facts and contribute to decision-making by: helping to recognise what needs to be thought about; identifying possible options; appraising options and making a selection; and supporting how patients cope with their decisions. Our findings indicate that health practitioners would benefit from more clarity and support about what counts as evidence, and how to synthesise these different types of evidence together in a way that provides the patients with all of the necessary skills they need to be able to make informed decisions about their healthcare.

Patients’ growing confidence in their own knowledge to make decisions about health information, and in turn their increasing expectations of healthcare, appeared to have a detrimental effect on some health practitioners’ confidence to communicate evidence. On examination, our participants, many of whom had more than ten years of professional experience, attributed their own confidence issues to a lack of knowledge about appraising evidence, which could leave them feeling deflated and embarrassed when challenged by seemingly knowledgeable patients. Wilson et al. [46] similarly found that nurses working in GP practices lacked the confidence to deal with patients who had high levels of knowledge. A recent Norwegian study found that nurse practitioners rarely use research and rely on other sources of information such as: their own and their colleagues’ practical knowledge; knowledge gained from their nursing education; nursing literature; and guidance from experts [47]. Within the UK community nursing context, research found that health visitors did report reading nursing journals but were more likely to read editorials and news articles than research articles [23]. Changes in healthcare policy such as a focus on multidisciplinary teams with specialisms, and more research and training opportunities, may alleviate some of the issues that led some of our participants to lack confidence in communicating evidence. However, research suggests that blurring of professional boundaries and job roles, which these reforms bring, can cause confusion for both patients and practitioners about what is expected from them [48,49]; thus potentially compounding health practitioners’ confidence issues.

The therapeutic relationship is often said to be at the heart of effective nursing practice and involves using a patient-centred approach to build respect and trust between the patient and the practitioner [26,50-52]. Commonly, our participants recognised that developing trust and a non-directive approach were essential components of communicating evidence effectively with their patients. However, some of our participants found it challenging to balance their delicate longer-term relationships with patients alongside communicating evidence-based information, and as a result would sometimes choose not to communicate evidence. Our participants were also concerned that not having the necessary information to hand would potentially undermine patient trust. These findings highlight that health practitioners are aware of the importance of building patient trust to enable them to engage in more meaningful conversations with patients about evidence, but that keeping up with the knowledge and skills needed to deliver evidence-based practices can be challenging. Ford et al. [18] have provided guidance on the types of relationship building and more technical evidence-based delivery skills that can help to foster evidence-based patient care. Some of the relationship building skills Ford et al. proposed to be helpful in strengthening the practitioner-patient relationship are: listening abilities; good eye contact; picking up on emotional and non-verbal cues; building up good rapport; letting the patient set the pace; appropriate reassurance; summarising and so on. Some useful evidence-based delivery skills proposed were: being able to simplify complex information; tailoring information to the patient’s needs and preferences; weighing up evidence and treatments; explaining probable risk; facilitating skills to encourage patient involvement; the evaluation of Internet information that patients may bring and so forth. The findings from the current research suggest that the health practitioners were very aware of the importance of building rapport and trust with their patients. However, they found it challenging to convey information about research evidence confidently to patients when they themselves were unsure of the evidence base, or when they did not feel they possessed all the necessary skills and knowledge. As a result, this hindered efforts to communicate evidence-based patient care as outlined by Ford and colleagues.

### Limitations

A potential limitation of this research is that the health visitors and practice nurses who took part in this study were self-selected and may represent a highly engaged sub-section of their professions. One obvious drawback to using telephone interviews is the absence of non-verbal cues, which can make this method more difficult. However, the researcher carrying out the interviews was experienced in this technique. There are also some contextual differences between the roles and settings in which health visitors and practice nurses work (aforementioned in the methods section) that could have affected the relationships that these two groups of health professionals have with their patients, although this was not evident in the data. It is also important to recognise that small-scale qualitative studies, such as this one, provide contextually bound in-depth accounts and as such are not necessarily generalisable to other contexts or groups. As previously mentioned, our data was collected in 2008/2009 and UK healthcare policy and training has undergone substantial changes in recent years. For example, undergraduate and postgraduate university education increasingly form a core aspect of nursing and midwifery training, and new policies have led to changes in the roles, responsibility, expertise and professional boundaries of multidisciplinary healthcare teams [35,53]. However, issues relating to ‘unacceptable variation’ in the quality of everyday care continue to pose challenges around specific areas, such as the need for nurses and midwives to be technically competent, well educated and patient-driven [35].

## Conclusions

This research highlights some of the emerging challenges that health practitioners face around communicating evidence in practice. Although the overall responsibility of evidence implementation is shared across teams, organisations and wider policy initiatives, frontline practitioners need to have the skills, knowledge and confidence to ensure they can effectively communicate about evidence with their patients. Our findings suggest that increasing public access to complex, and sometimes contradictory, health information alongside higher patient expectations may result in growing demands and challenges being placed upon busy health practitioners. How health practitioners deal with and respond to these challenges may reflect how confident they feel in their own abilities to assess and communicate about research evidence and the type of relationship they build with their patients. In the UK, healthcare reforms in nursing and midwifery aspire to increase educational training and there are a greater number of opportunities for continued professional development for practitioners to learn about appraising and communicating evidence. Reforms have also led to greater interdisciplinary team working, which may improve access to expertise and support.

Health practitioners in this study often found it challenging to know how to contextualise best evidence with conflicting information, patients’ preferences and wider social and political influences that shape patients’ attitudes and beliefs. As a result, in some instances where patients were entrenched in their views, practitioners would ‘weigh up’ these various factors and decide not to advise patients about the evidence in fear of jeopardising their valued longer-term relationships. Community-based health practitioners should seek to combine their relationship-building skills and information giving skills to enter into more meaningful and engaged conversations with patients about evidence and how specific treatments and care would fit in with their patients’ values and preferences.

One of the big challenges that healthcare continues to face is how to operationalise evidence-based practice. If health practitioners are to be effective at communicating research evidence in the current healthcare system, we suggest that more research and resources need to be focused on contextual factors such as understanding how research evidence is negotiated, appraised and communicated within the dynamic patient-practitioner relationship.

## Competing interests

The authors declare that they have no competing interests.

## Authors’ contributions

JvB participated in the analysis and drafting of the manuscript. SH participated in the design, data collection, analysis, and in drafting the manuscript. Both authors approved the final manuscript.

## Pre-publication history

The pre-publication history for this paper can be accessed here:

http://www.biomedcentral.com/1472-6955/12/17/prepub
